# Workplace interventions that aim to improve employee health and well-being in male-dominated industries: a systematic review

**DOI:** 10.1136/oemed-2020-107314

**Published:** 2021-05-25

**Authors:** Paige M Hulls, Rebecca C Richmond, Richard M Martin, Yanaina Chavez-Ugalde, Frank de Vocht

**Affiliations:** 1 Population Health Sciences, Bristol Medical School, University of Bristol, Bristol, UK; 2 MRC Integrative Epidemiology Unit, University of Bristol, Bristol, UK; 3 NIHR Bristol Biomedical Research Centre, University Hospitals Bristol NHS Foundation Trust and the University of Bristol, Bristol, UK; 4 National Institute for Health Research, School for Public Health Research, Newcastle upon Tyne, UK; 5 National Institute for Health Research Collaboration for Leadership, Applied Health Research and Care West (NIHR CLAHRC West), Bristol, UK

**Keywords:** occupational health, health personnel, mental health, occupational stress

## Abstract

The published evidence on whether workplace health and well-being interventions are as effective in male-dominated industries compared with mixed-gender environments has not been synthesised. We performed a systematic review of workplace interventions aimed at improving employee health and well-being in male-dominated industries. We searched Web of Knowledge, PubMed, Medline, Cochrane Database and Web of Science for articles describing workplace interventions in male-dominated industries that address employee health and well-being. The primary outcome was to determine the effectiveness of the intervention and the process evaluation (intervention delivery and adherence). To assess the quality of evidence, Cochrane Collaboration’s Risk of Bias Tool was used. Due to the heterogeneity of reported outcomes, meta-analysis was performed for only some outcomes and a narrative synthesis with albatross plots was presented. After full-text screening, 35 studies met the eligibility criteria. Thirty-two studies delivered the intervention face-to-face, while two were delivered via internet and one using postal mail. Intervention adherence ranged from 50% to 97%, dependent on mode of delivery and industry. 17 studies were considered low risk of bias. Albatross plots indicated some evidence of positive associations, particularly for interventions focusing on musculoskeletal disorders. There was little evidence of intervention effect on body mass index and systolic or diastolic blood pressure. Limited to moderate evidence of beneficial effects was found for workplace health and well-being interventions conducted within male-dominated industries. Such interventions in the workplace can be effective, despite a different culture in male-dominated compared with mixed industries, but are dependent on delivery, industry and outcome. CRD42019161283.

**Key messages**

**What is already known about this subject?**

- Male-dominated industries have a higher prevalence of risky health behaviours and masculine norms can contribute to poorer health outcomes in men.
- Systematic reviews in this field have focused on specific theme, instead of comparing the different areas of employee health and well-being.

**What are the new findings?**

- The albatross plots indicated evidence of positive associations, particularly for musculoskeletal disorders.
- Intervention adherence ranged from 50% to 97%, dependent on mode of delivery and industry, and 32 of the 35 included studies delivered the intervention face-to-face.

**How might this impact on policy or clinical practice in the foreseeable future?**

- Improving employee health and well-being with workplace interventions is possible, but intervention content and delivery must be considered.
- Researchers need to consider an organisational rather than individual approach to have a beneficial effect on employees’ health and well-being.

## Background

Beyond providing income to meet basic needs, being employed can benefit health by providing meaningful activity and structure to the day, opportunities for social contact, and making up a key part of one’s social identity.^1^ The WHO defines a healthy workplace as ‘one in which workers and managers collaborate to use a continual improvement process to protect and promote the health, safety and well-being of all workers and the sustainability of the workplace’.^2^ However, certain work activities can put employees’ health at risk in the form of occupational risks and hazards, as well as the impact on their mental health.

Male-dominated industries are commonly defined as comprising over 70% male workers, and include agriculture, construction, manufacturing, mining, transport and technology.^1^ Masculine norms, ‘culturally accepted rules and standards that guide and constrain masculine behaviours’, may also contribute to poorer health outcomes of both men and women in male-dominated occupations.^3^ Due to the nature of the work, employees in male-dominated industries have an elevated risk of work-related injuries and fatalities,^3^ while they also have a higher prevalence of poor health outcomes (in men and women) compared with gender-balanced industries.^4^ The combination of poor physical and psychological working conditions is thought to partially explain the higher prevalence of risky health behaviours and elevated disease burden in male-dominated industries.^3^

Workplace health interventions offer an opportunity to reach a significant proportion of the working population. Interventions have emerged as a set of comprehensive health promotion and occupational health strategies implemented at the workplace to improve work-related outcomes.^5^ There is a strong case for employers to engage in employee health and well-being programmes, alongside legal obligations and corporate social responsibility. Benefits at the organisational level include increased productivity, improved employee retention, reduced sickness absence and greater employee resilience.^6^

While systematic reviews on interventions in the male-dominated industries have been conducted, they have focused on one specific theme (eg, mental health, physical activity and smoking cessation) instead of comparing the different areas of employee health and well-being.^1^ Our aim was to systematically review workplace interventions aimed at improving employee health and well-being specifically in male-dominated industries and quantify their effectiveness.

## Methods

### Patient and public involvement

No patients were involved.

### Protocol and registration

This systematic review was registered with the International Prospective Register of Systematic Reviews (PROSPERO; registration number CRD42019161283; available from https://www.crd.york.ac.uk/prospero/display_record.php?ID=CRD42019161283). It was conducted and reported following the Preferred Reporting Items for Systematic Reviews and Meta-Analyses (PRISMA) statement for reporting systematic reviews and meta-analyses.^7^

### Eligibility criteria

Studies had to have been written in the English language and published in a peer-reviewed journal. Grey literature, including conference abstracts, abstracts and dissertations, were not considered. Studies were not excluded if they were developed and/or launched by employers and then effectiveness was evaluated by research teams. Studies had to have quantitatively evaluated an intervention implemented in the workplace and which aimed to alter the health behaviours of employees. Study designs were either a randomised controlled trial (RCT) or a non-randomised intervention group allocation. Only studies in industries with male-dominated employee populations were included. Assignment of male-dominated industries was a priori based on the industrial sector: construction, manufacturing, mining, transport, agriculture and technology.^1^ Studies had to include information and measures of physical or psychological health and risk behaviours that may affect, or be the result of, physical or psychological health issues, that is, blood pressure, weight, alcohol consumption or mental well-being. Further details on the methodology have been described in the study protocol.^8^

### Search strategy

Five electronic databases were searched to identify articles published up to 26 October 2020: Web of Knowledge, PubMed, Medline, Cochrane Database and Web of Science. The search strategy used medical subject headings (MeSH) and keywords (eg, male-dominated, stress, employee, intervention). Specific search strategies are outlined in online supplemental figure 2. The reference lists of the articles that were included in the final review were screened for additional eligible articles that the online bibliographic database search had missed. After the initial search, references were imported into EndNote to remove duplicates (identified by title, author and DOI). Following the PROSPERO and PRISMA guidelines, titles and abstracts of identified articles were screened, followed by full-text screening, conducted by one of the authors (PH). The titles and abstracts of selected studies were also independently screened by a second author (YC-U) and discrepancies between the authors were discussed until consensus was reached.

10.1136/oemed-2020-107314.supp1 Supplementary data

### Data extraction

The criteria for data extraction were determined prior to starting the review. The primary outcomes of interest were effectiveness of the intervention (as defined by the individual study author), intervention delivery, intervention adherence, measures of physical health and measures of psychological health or occupational stress using validated scales. Summary data from each study were collected into a standardised, predetermined form and included study design, participants, setting (workplace industry), intervention details (type and content), outcomes (pre, post and follow-up) and acceptability or participant satisfaction relating to the intervention. While we included studies that focused on job strain, we chose not to use the definition based on Karasek’s model to ensure that we were able to include all interventions that were aimed to impact on job strain itself, as defined in the Karasek’s model, or on intermediate biological factors closely related to job strain.

### Quality assessment

Study methodologies were assessed using the Cochrane Collaboration’s Risk of Bias Tool for randomised and non-randomised interventions. Studies were categorised by PH into low risk of bias, unclear risk of bias or high risk of bias, based on the following criteria: selection bias, reporting bias, performance bias, detection bias, attrition bias and other bias.

### Data analysis

Due to the heterogeneity of interventions, measures and outcomes reported by the included studies, the findings are presented in narrative synthesis incorporating effect sizes and CIs where reported, or p values where these were not provided. From the 35 studies, 54 objective and 61 subjective (self-reported) outcomes were included. Of the 107 outcomes, 16 outcomes were reported across multiple studies. For studies measuring body mass index (BMI) and blood pressure, forest plots were constructed to examine underlying effect sizes from the workplace interventions and the heterogeneity across the studies. I^2^ statistics were used to assess between-study heterogeneity. An albatross plot was created to display the direction of the observed effects of all included interventions. The plot is a graphical tool that allows the presentation of results of diversely reported studies in a systematic review.^9^ All analyses were conducted using Stata V.15.^10^

## Results

**Figure 1:**
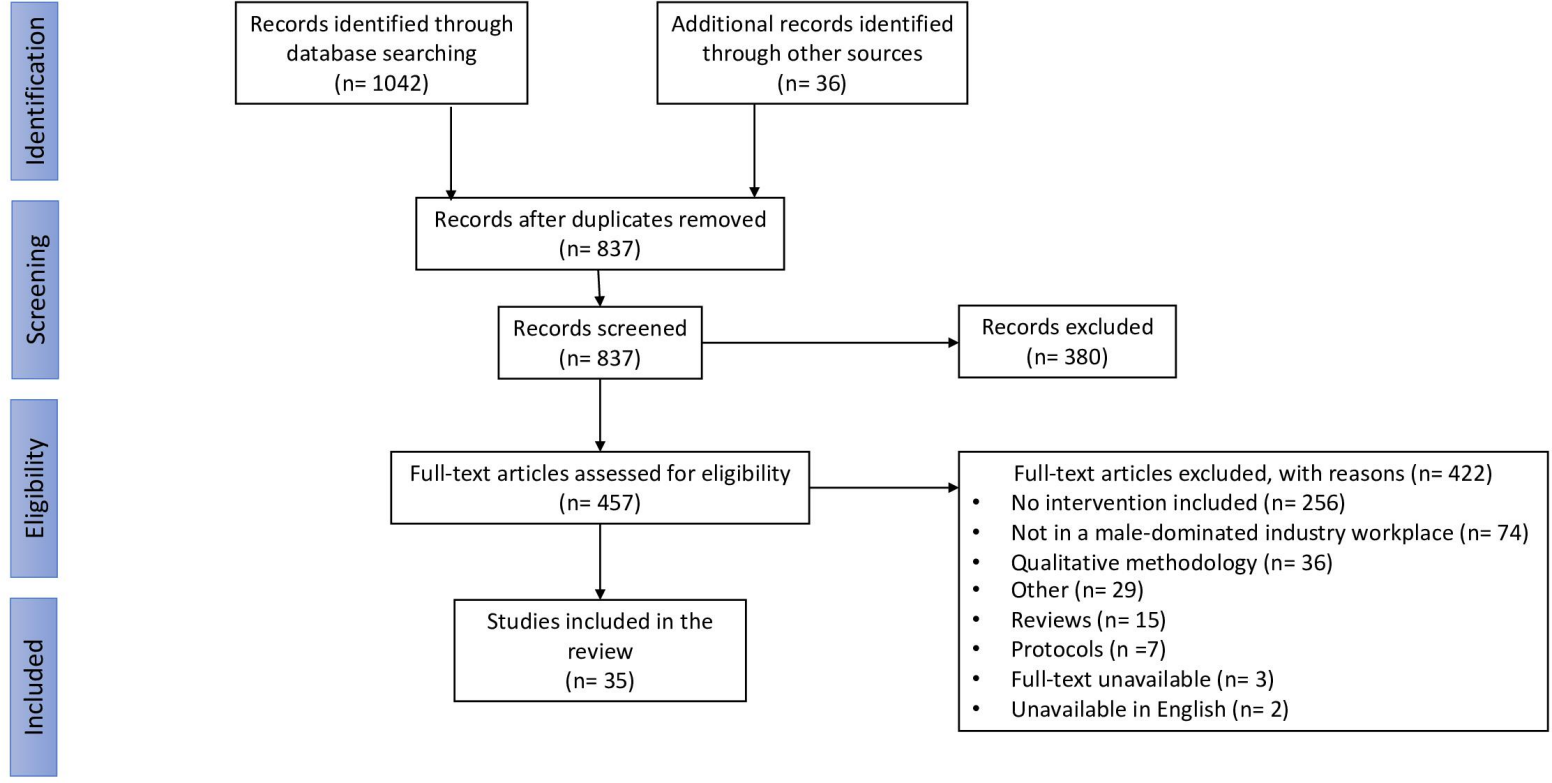
PRISMA flow diagram. PRISMA, Preferred Reporting Items for Systematic Reviews and Meta-Analyses.

The initial search resulted in a total of 837 articles, not including duplicates. Based on titles and abstracts, 435 full texts were retrieved for a full review. Of these, a further 407 were excluded. A PRISMA flow diagram displaying the search results can be found in figure 1.

The studies were conducted in Asia (one Indian, seven Japanese, one Chinese, one South Korean), Europe (six Dutch, four Danish, two Swedish, one Belgian, one Swiss, one Spanish, one Italian, one German, one Polish), North America (three American), South American (one Chilean) and Australasia (three Australian). Of the studies, 13 were in the construction sector, 12 in the manufacturing sector, 4 in the information technology (IT) sector, 1 in the transport sector and 1 in the mining sector.

Based on the intervention aim, nine health and well-being domains were identified: job strain (n=8), musculoskeletal disorders (n=7), lifestyle (n=5), diet and physical activity (n=4), physical activity (n=4), nutrition (n=2), smoking cessation (n=2), mental health (n=2), alcohol (n=1), depression (n=1) and sleep (n=1).

The length of the interventions varied from 2 weeks to 3 years, with 34% of studies having a 6-month intervention and the average intervention length being 28 weeks.

The review includes both male and female workers in male-dominated industries. Of the 35 studies included in the systematic review, 13 studies had male-only samples^11–24^ and 2 studies had female-only samples.^20 25^ Of the 20 studies with a mixed sample, only 3 provided information regarding gender-specific effects of the intervention.^26–28^

In male workers there was a negative intervention effect for intrinsic reward (p=0.040) and in female workers there was a favourable intervention effect for depression and vigour (p<0.05), working in Japanese manufacturing companies. There was no significant effect observed for sick leave in either gender.^27^ In the same population,^26^ there was no significant intervention effect (p>0.05) on job strain, via mailed advice, on analysis of subgroups classified by gender. A lifestyle intervention in Dutch manufacturing employees had a favourable intervention effect on serum cholesterol levels in male (p=0.02) and female (p=0.09) workers.^28^

Table 1 summarises each study and online supplemental table 1 provides a summary of their main findings.

**Table 1:** Summary of characteristics of studies that included workplace interventions aiming to improve employee health and well-being in male-dominated industries

Author (year)	Design	Industry	Participants, context/setting/% of men	Intervention	Outcomes	Follow-up time points	Risk of bias	Intervention evaluation
Anderson and Dusenbury (1999)^32^	Quasi-experimental; participants assigned to intervention 1 (n=61), intervention 2 (n=35) and control (n=118).	Construction.	234 blue-collar employees from worksites/USA/52.5%.	Intervention 1: group education classes for risk factors, nutrition. Intervention 2: online individual programme for risk factors, nutrition.	Cholesterol; blood pressure; height; weight; behavioural risk factor survey; 10-item questionnaire.	Baseline, 6 and 12 months.	Unclear.	Local government and research study.
Blake *et al* (2019)^31^	Cluster RCT; participants assigned to the intervention (n=196) and control (n=86).	Information technology.	282 office workers/China/50.3%.	10 min Qigong exercise session delivered twice per day at set times for 12 weeks via video.	Physical activity (IPAQ); work performance (WHO HPQ); weekday sitting hours.	Baseline and 12 weeks.	Low.	Research study.
Braeckman *et al* (1999)^11^	RCT; participants assigned to the intervention (n=272) and control (n=366).	Construction.	638 workers from four local worksites/Belgium/100%.	Adopt a low-fat diet. Received personal counselling, feedback and 2-hour group session.	BMI; high-density cholesterol; non-fasting total serum cholesterol; 24-hour food record.	Baseline and 3 months.	Unclear.	Research study.
Evans *et al* (1999)^40^	Controlled trial; participants assigned to the intervention (n=10) and control (n=31).	Transport.	41 full-time bus operators/Sweden/67.5%.	Changes on a major bus route: separate bus lanes, priority traffic signal system.	Blood pressure; heart rate; Swedish measure of perceived stress; on-the-job hassles.	Baseline and 18 months.	High.	Local government and research study.
Faude *et al* (2015)^33^	Longitudinal controlled trial; participants assigned to the intervention (n=45) and control (n=25).	Construction.	70 employees from one construction company/Switzerland/ND.	Neuromuscular training for 15 min daily for 13 weeks.	COP path length; beam balancing (3 cm); beam balancing; jump height; Freiburg Physical Activity Questionnaire.	Baseline, 8 and 13 weeks.	Unclear.	Research study.
Gram *et al* (2012)^12^	RCT; participants assigned to the intervention (n=35) and control (n=32).	Construction.	67 employees from three construction companies/Denmark/100%.	Exercise programme of 3×20 min sessions per week. Participants kept a training log.	Pain intensity; work ability; productivity; perceived physical exertion; sick leave.	Baseline and 6 months.	Low.	Research study.
Gram *et al* (2012)^13^	RCT; participants assigned to the intervention (n=35) and control (n=32).	Construction.	67 employees from three construction companies/Denmark/100%.	Exercise programme of 3×20 min sessions per week. Participants kept a training log.	Pain intensity; work ability; productivity; perceived physical exertion; sick leave.	Baseline and 6 months.	Unclear.	Research study.
Groeneveld *et al* (2010)^15^	RCT; participants assigned to the intervention (n=408) and control (n=408).	Construction.	816 construction workers/the Netherlands/100%.	3×60 min and 4×30 min sessions. Chose diet, physical activity or smoking cessation.	Body weight; BMI; systolic and diastolic blood pressure; HDL cholesterol; total cholesterol; HbA1c.	Baseline 6 and 12 months.	Low.	Research study.
Groeneveld *et al* (2011)^14^	RCT; participants assigned to the intervention (n=408) and control (408).	Construction.	816 construction workers/the Netherlands/100%.	3×60 min and 4×30 min sessions. Chose diet, physical activity or smoking cessation.	Body weight; BMI; systolic and diastolic blood pressure; HDL cholesterol; total cholesterol; HbA1c.	Baseline, 6 and 12 months.	Low.	Research study.
Gupta *et al* (2018)^34^	Cluster RCT; participants assigned to the intervention (n=193) and control (n=122).	Manufacturing.	415 employees from three manufacturing plants/Denmark/70.4%.	Participated in visual mapping talk with line management. Leaders, union and H&S representatives also participated.	Worker’s recovery; work ability; mental health; well-being; physical work demands; resources; productivity.	Baseline, 10 and 12 months.	Low.	Research study.
Hammer *et al* (2015)^41^	RCT; participants assigned to the intervention (n=167) and control (n=125).	Construction.	264 employees from an urban municipal department/USA/90%.	Supervisors participated in FSSB and SBS computer-based training. All employees participated in workshop planning.	Blood pressure; SF-12; safety behaviours.	Baseline and 12 months.	Unclear.	Research study.
Holmström and Ahlborg (2005)^16^	Cluster RCT; participants assigned to the intervention (n=30) and control (n=17).	Construction.	57 employees from a construction company/Sweden/ND.	10 min exercises before work for 3 months.	Neck mobility; spine mobility; shoulder joint mobility.	Baseline and 3 months.	Unclear.	Research study.
Kang *et al* (2018)^35^	RCT; participants assigned to the intervention (n=12) and control (n=12).	Manufacturing.	24 employees from an automobile assembly plant/South Korea/ND.	Performed exercises for 30 min each day for 6 weeks.	Back muscle strength; stork balance stand test; VAS; Oswestry Disability Index; Beck Depression Inventory.	Baseline and 6 weeks.	Unclear.	Research study.
Kawakami *et al* (1999)^26^	RCT; participants assigned to the intervention (n=81) and control (n=77).	Manufacturing.	158 employees from a manufacturing plant/Japan/81%.	Participants received personalised letters with their stress levels and recommendations to help improve.	GHQ; blood pressure; serum cholesterol; triglycerides; sick leave.	Baseline and 12 months.	Unclear.	Research study.
Kobayashi *et al* (2008)^27^	Controlled trial; participants assigned to the intervention (n=348) and control (n=918).	Manufacturing.	1266 employees from a manufacturing plant/Japan/92.9%.	Participated in a mental health workshop, identify three actions to improve the workplace.	Absenteeism; BJSQ; job stress assessment diagram.	Baseline and 12 months.	Low.	Company and research study.
Limaye *et al* (2017)^30^	RCT; participants assigned to the intervention (n=133) and control (n=132).	Information technology.	266 employees from two information technology industries/India/74.8%.	Attended group session and OW/OB participants aimed to lose 5% weight via four goals.	BMI; waist circumference; blood pressure; plasma glucose; triglyceride; total cholesterol; HDL cholesterol; lifestyle questionnaire.	Baseline, 3, 6, 9 and 12 months.	Low.	Research study.
Limm *et al* (2011)^42^	RCT; participants assigned to the intervention (n=75) and control (n=79).	Manufacturing.	174 employees from a manufacturing plant/Germany/99%.	Participated in 24×45 min group sessions on individual work stress situations.	Stress reactivity scale; effort-reward imbalance model; cortisol; α-amylase; HADS.	Baseline and 12 months.	Low.	Research study.
Maes *et al* (1998)^28^	Quasi-experimental; participants assigned to the intervention (n=234) and control (n=130).	Manufacturing.	264 employees from Brabantia assembly sites/the Netherlands/ND.	Year 1: lifestyle changes with three weekly sessions. Years 2 and 3: quality of work changes with leadership session.	BMI; heart rate; systolic and diastolic blood pressure; total cholesterol; absenteeism; wellness at work; Symptom Checklist-90; Work Stress Questionnaire.	Baseline, years 1, 2 and 3.	Unclear.	Company and evaluated as a research study.
Matsugaki *et al* (2019)^39^	RCT; participants assigned to the intervention (n=30) and control (n=30).	Manufacturing.	60 employees from manufacturing companies/Japan.	Monthly, face-to-face personalised physical activity and nutrition education programme for 6 months.	30 s chair stand; grip strength; balance; body composition.	Baseline and 6 months.	Low.	Research study.
McCraty *et al* (2003)^43^	RCT; participants assigned to the intervention (n=18) and control (n=14).	Information technology.	38 employees from an information technology company/America/71.5%.	Participated in a 16-hour programme addressing positive emotion refocusing and emotional restricting techniques.	Blood pressure, emotional health; workplace-related measures.	Baseline and 3 months.	Unclear.	Company and evaluated as a research study.
Milner *et al* (2018)^18^	RCT; participants assigned to the intervention (n=343) and control (n=302).	Construction.	682 employees from construction companies/Australia/100%.	Access to the Contact+Connect programme with 1 weekly message for 6 weeks.	Self-Stigma of Depression Scale; suicidal ideation; suicide communication; SBQ-R.	Baseline and 6 weeks.	Low.	Company and evaluated as a research study.
Milner *et al* (2020)^17^	RCT; participants assigned to the intervention (n=227) and control (n=215).	Construction.	442 employees from construction companies/Australia/100%.	Access to the Contact+Connect programme with 1 weekly message for 6 weeks.	Self-Stigma of Depression Scale; suicidal ideation; suicide communication; SBQ-R.	Baseline and 6 weeks.	Low.	Company and evaluated as a research study.
Molek-Winiarska and Żołnierczyk-Zreda (2018)^19^	RCT; participants assigned to the intervention (n=32) and control (n=34).	Mining.	66 employees from a mining company/Poland.	Received the MBSR intervention, sessions were held in 4×8-hour meetings and one mindfulness days.	JCQ; GHQ-28.	Baseline and 3 months.	Unclear.	Research study.
Muñoz-Poblete *et al* (2019)^38^	RCT; participants assigned to the intervention (n=53) and control (n=56).	Manufacturing.	109 employees from manufacturing companies/Chile/80.8%.	Received a resistance-based exercise programme, 3 times a week for 15 min. Control group received stretching exercises.	VAS; DASH questionnaire; psychosocial risk measurement; physical risk measurement.	Baseline and 16 weeks.	Unclear.	Research study.
Muyor *et al* (2012)^20^	RCT; participants assigned to the intervention (n=27) and control (n=31).	Manufacturing.	58 employees from a manufacturing company/Spain/0%.	Hamstring stretches/exercises three times a week for 12 weeks.	Straight leg raise (right and left leg); toe-touch test.	Baseline and 12 weeks.	Unclear.	Research study.
Nakao *et al* (2007)^21^	Cohort; participants assigned to the intervention (n=283) and control (n=22).	Information technology.	305 employees from an information technology company/Japan/100%.	Offered counselling via email/phone and referred to psychiatric clinic. Attended five seminars.	HAM-D and JCQ.	Baseline and 2 years.	Unclear.	Company and research study.
Nishinoue *et al* (2012)^44^	RCT; participants assigned to the intervention (n=62) and control (n=62).	Information technology.	127 employees from an information technology company/Japan/85.75%.	Sleep hygiene education session followed by individual session discussing their chosen behaviour modification.	PSQI.	Baseline and 3 months.	Unclear.	Research study.
Oude Hengel *et al* (2012)^22^	RCT; participants assigned to the intervention (n=171) and control (n=122).	Construction.	293 employees from six construction companies/the Netherlands/99%.	Individual training sessions with three physical goals. Two group mental health sessions.	JCQ; Utrecht Work Engagement Scale; physical workload; VBBA.	Baseline, 3, 6, 9 and 12 months.	Low.	Research study.
Oude Hengel *et al* (2013)^23^	RCT; participants assigned to the intervention (n=171) and control (n=122).	Construction.	293 employees from six construction companies/the Netherlands/99%.	Individual training sessions with three physical goals. Two group mental health sessions.	Absenteeism; Work Ability Index; SF-12; Dutch Musculoskeletal Questionnaire.	Baseline, 3, 6, 9 and 12 months.	Low.	Research study.
Pidd *et al* (2018)^36^	Cluster non-RCT; participants assigned to the intervention (n=169) and control (n=148).	Manufacturing.	317 employees from three manufacturing companies/Australia/87.4%.	Formal alcohol workplace policy, employee programme, manager training session and employee referral pathway.	3-item AUDIT-C; European alcohol workplace and alcohol baseline questionnaire; alcohol-related harm in the workplace; policy awareness.	Baseline and 12 months.	Unclear.	Company and research study.
Rasotto *et al* (2015)^25^	Cluster RCT; participants assigned to the intervention (n=30) and control (n=30).	Manufacturing.	60 employees from a manufacturing company/Italy/0%.	Exercise programme for 30 min twice a week for 6 months.	VAS (neck, elbow, shoulder, wrist); SH (el, ab); FL head; EX head; LI head; RO head; DASH questionnaire; NPDS-1.	Baseline and 6 months.	Low.	Research study.
Umanodan *et al* (2009)^45^	Controlled trial; participants assigned to the intervention (n=96) and control (n=53).	Manufacturing.	149 employees from a steel company/Japan/90%.	6-monthly sessions lasting 30 min following a multicomponent SMT programme.	BJSQ; MBI-GS; WHO HPQ.	Baseline and 6 months.	High.	Research study.
Umanodan *et al* (2014)^46^	Cluster RCT; participants assigned to the intervention (n=142) and control (n=121).	Manufacturing.	263 employees from a manufacturing company/Japan/92.6%.	Computer-based SMT intervention, each split into two sessions. Suggested pace of 1 per week.	BJSQ; UWES-J; WHO HPQ; BSCP.	Baseline, 9 and 19 weeks.	Low.	Research study.
Viester *et al* (2018)^24^	RCT; participants assigned to the intervention (n=162) and control (n=152).	Construction.	314 employees from construction companies/the Netherlands/100%.	Individual coaching sessions to change lifestyle behaviour over 6 months.	BMI; waist circumference; blood pressure; total cholesterol; SQUASH questionnaire.	Baseline, 6 and 12 months.		Research study.
Zebis *et al* (2011)^37^	RCT; participants assigned to the intervention (n=282) and control (n=255).	Manufacturing.	537 employees from two manufacturing companies/Denmark/15.5%.	Exercise programme for 1 hour per week over 20 weeks.	Modified Nordic Questionnaire; training frequency.	Baseline and 6 weeks.	Low.	Research study.

AUDIT-C, Alcohol Use Disorders Identification Test - Consumption; BSCP, Brief Scales for Coping Profile; FSSB, Family-Supportive Supervisor Behaviours; HADS, Hospital Anxiety and Depression Scale; HAM-D, Hamilton Depressing Rating Scale; HbA1c, Haemoglobin A1c; H&S, Health and Safety; MBI-GS, Maslach Burnout Inventory - General Survey; MBSR, Mindfulness-Based Stress Reduction; PSQI, Pittsburgh Sleep Quality Index; SBS, Supervisor Based Safety; SH (el, ab), Shoulder (elevation, abduction); SMT, Stress Management Training; UWES-J, Utrecht Work Engagement Scale - Japanese.

### Risk of bias

**Figure 2:**
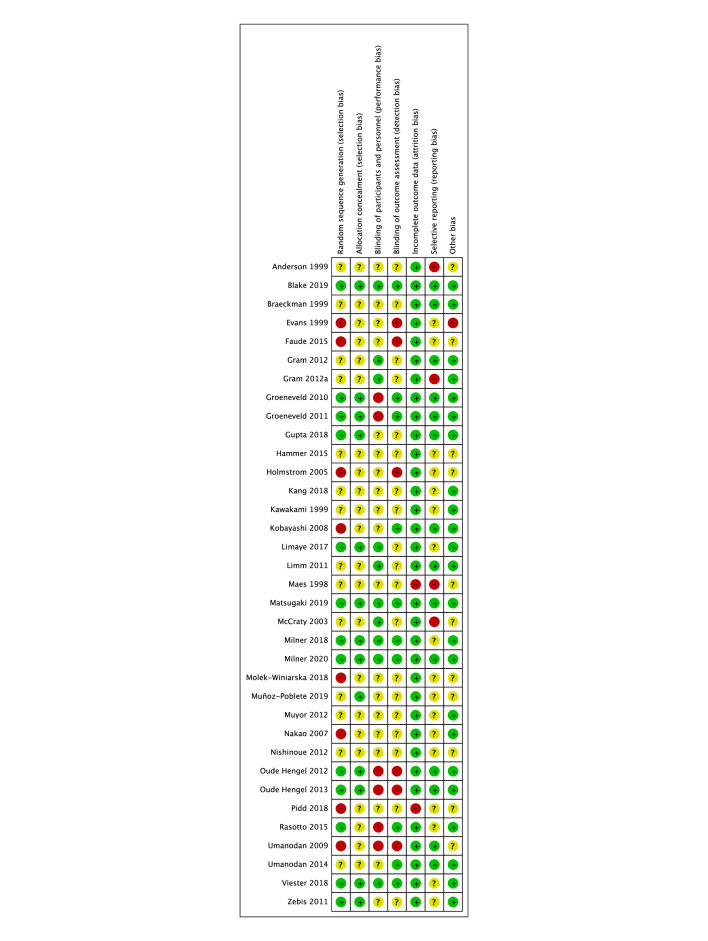
Risk of Bias Tool.

Of the 35 studies, 17 were considered to have a low risk of bias, 16 had an unclear risk of bias and 2 had a high risk of bias (figure 2). As only two studies had a high risk of bias, they were not excluded from any results analysis. Studies whose intervention focus included lifestyle, diet and physical activity, physical activity, smoking cessation, and mental health were rated to be at low risk of bias overall. In comparison, reporting in studies that aimed to address job strain, musculoskeletal disorders, nutrition, alcohol, depression and sleep was less comprehensive and they were rated as having unclear risks of bias.

### Intervention delivery

The majority of studies (32) implemented the intervention face-to-face, while two studies delivered the intervention using the internet in IT,^29–31^ and one study sent intervention content to participants via postal mail^26^ in the manufacturing industry. Of the studies using face-to-face delivery, 13 were conducted in the construction industry,^11–18 22 23 27 32 33^ 10 in the manufacturing industry,^18 20 24 25 28 34–39^ 1 in the mining industry^19^ and 1 in the transport industry.^40^

### Intervention adherence

Intervention adherence, defined by the individual study authors, across studies ranged from 50% to 97%. Adherence to internet interventions ranged between 50% and 78%^29 30^; when using postal mail adherence was 89%,^26^ while face-to-face intervention adherence was between 57% and 99%.^11–19 21–25 27 28 32–34 36–39 41–46^ Four studies did not disclose adherence rates.^20 31 35 40^ Within the construction industry, intervention adherence ranged from 57% to 94%,^11–18 22–24 27 32 33 41^ while in the manufacturing industry intervention adherence was 50%–99%,^20 25 26 28 29 34–39 42 45 46^ 78%–95% for studies within the IT industry^21 30 43 44^ and 97% for the intervention in the mining industry.^19^

### Measures of physical health

Studies commonly used objective measures of physical health as their primary or secondary outcomes, including cholesterol, blood pressure, heart rate, BMI and waist circumference. Nine studies were in the construction sector,^11–15 23 24 32 41 47^ three in the manufacturing sector,^26 28 39^ two in the IT sector^30 43^ and one in the transport sector.^40^ There were four studies focusing on diet and physical activity,^14 15 24 30^ three studies focused on job strain,^26 40 43^ three studies focused on lifestyle,^23 28 41^ two studies focused on nutrition^11 32^ and three studies focused on physical activity.^12 13 39^

Blood pressure was assessed as an outcome in 10 studies. Five studies reported an intervention effect on blood pressure, two of which focused on job strain,^40 43^ two focused on lifestyle^28 30 41^ (as defined by the individual study authors) and one focused on diet and physical activity.^15^ For the two studies focusing on job strain, one reported an intervention effect on systolic blood pressure with p<0.01^40^ and one reported an average reduction of 9.0±3.0 mm Hg in one study^43^ and p<0.01 in another,^40^ but no effects for systolic or diastolic blood pressure. For the two studies focusing on lifestyle, the intervention reduced systolic blood pressure by −1.9 mm Hg (95% CI −3.2 to –0.6; p=0.45) and diastolic blood pressure by −1.3 mm Hg (95% CI −2.3 to –0.3; p=0.03) at 6 months, but not at 12 months,^31^ while Hammer *et al* 41 reported an intervention effect on mean blood pressure of −2.2 mm Hg (95% CI −2.32 to 2.89; p<0.038) at 12 months. A diet and physical activity intervention lowered diastolic blood pressure versus the control group (−1.7 mm Hg, 95% CI −3.3 to –0.1; p<0.05) at 6 months, but not systolic blood pressure (−2.2 mm Hg, 95% CI −4.6 to 0.3).

No interventions effects were reported for four studies focusing on nutrition,^32^ physical activity,^13^ job strain^26^ and diet and physical activity,^24^ respectively. After a 12-week physical activity intervention, no evidence was reported for an effect on systolic blood pressure (2.9 mm Hg, 95% CI −4.9 to 6.6; p=0.77), diastolic blood pressure (2.4 mm Hg, 95% CI −3.2 to 6.4; p=0.51) and total cholesterol (0.2 mmol/L, 95% CI −0.1 to 0.4; p=0.56).^13^ Similarly, for interventions aimed at reducing job strain, there was little evidence of intervention effect for systolic blood pressure (p=0.93), diastolic blood pressure (p=0.31) and total cholesterol (p=0.23).^26^ There was little evidence at both 6 and 12 months of an effect from a diet and physical activity intervention on systolic blood pressure (−0.5 mm Hg, 95% CI −3.9 to 2.9; and 0.5 mm Hg, 95% CI −3.1 to 4.1) (p=0.77 and p=0.78), diastolic blood pressure (−0.05 mm Hg, 95% CI −2.3 to 2.2; and 2.0 mm Hg, 95% CI −0.4 to 4.5) (p=0.97 and p=1.02) and total cholesterol (0.03 mmol/L, 95% CI −0.2 to 0.2; and 0.07 mmol/L, 95% CI −0.1 to 0.2) (p=0.73 and p=0.40).^24^ For the intervention focusing on nutrition, there was no reported effect on either systolic or diastolic blood pressure and cholesterol.^32^

Eight studies measured BMI as an outcome,^11 13–15 24 30 32 39^ of which seven were RCTs and one had a quasi-experimental design.^32^ Six found that the workplace intervention resulted in an improvement in BMI in the intervention group compared with the control group. Two RCTs reported differing effects in the intervention group on BMI; one decreased and another increased BMI: −0.4 kg/m^2^ (95% CI −0.6 to –0.2)^30^ and 0.26 kg/m^2^ (95% CI 0.13 to 0.39),^11^ on average. Another two RCTs reported intervention effects on BMI (−0.6 kg/m^2^, 95% CI 0.8 to –0.3) both at 6 and 12 months, versus the control group.^14 15^ An RCT measuring 6 months and 12 months post intervention reported an intervention effect on BMI of −0.29 kg/m^2^ (95% CI −0.52 to –0.05; p=0.02) and −0.25 kg/m^2^ (95% CI −0.55 to 0.05; p=0.11),^24^ respectively. Two studies—one quasi-experimental design^42^ (data not provided) and the other an RCT^46^—did not report evidence of an intervention effect on BMI (0.1 kg/m^2^, 95% CI −0.3 to 0.6; p=0.55).

Several studies relied on questionnaires to measure physical health, including Short Form Health Survey (SF-12) and General Health Questionnaire. Using the SF-12 questionnaire, a diet and physical activity intervention for construction workers on work ability, health and sick leave found no intervention effects on either physical or mental health status (−0.04 points; 95% CI −1.43 to 1.35).^23^ Another RCT for construction and utility workers provided an intervention for work–life stress and safety-related psychosocial risk factors. While there was a reduction in blood pressure at 12 months (−2.15 mm Hg; p<0.05), there was no effect between control and intervention groups for mean SF-12 physical activity composite scores (−0.32 points; 95% CI −19.3 to 1.29).^41^

### Measures of psychological health or occupational stress

Seventeen studies used measures of psychological health or occupational stress for either primary or secondary outcomes. Of these, seven studies were in the manufacturing sector,^26 28 29 36 42 45 46^ seven studies were in the construction sector,^12 17 18 22 23 27 41^ two studies in the IT sector,^21 43^ one study in the transport sector^40^ and one study in the mining sector.^19^ The majority of these studies aimed to reduce job strain,^19 26 27 40 42 43 45 46^ lifestyle,^22 23 28 41^ mental health,^17 18^ depression,^21^ alcohol,^36^ physical activity^12^ and tinnitus distress.^29^

The most commonly used validated scales were the Brief Job Stress Questionnaire,^27 45 46^ Job Content Questionnaire (JCQ)^19 21 22^ and Utrecht Work Engagement Scale.^22 46^ A study with Japanese manufacturing employees^27^ concluded that there was little evidence that the implementation of an organisational intervention had an effect in men. However, for women, skill underutilisation (test value=3.9, 95% CI 2.09 to 2.71), supervisor and coworker support (test value=22.4, 95% CI 5.68 to 7.32; and test value=4.5, 95% CI 6.75 to 8.44) and psychological distress (test value=5.1, 95% CI 5.52 to 7.28) improved (p<0.05). While delivering a stress management training programme face-to-face over 6 months, one study reported a positive effect on knowledge (*F*=32.9, p<0.001).^45^ In comparison, an intervention delivered using a computer to improve psychological well-being and work performance in manufacturing employees had little effect on psychological distress.^46^

In a mindfulness-based stress reduction intervention for workers in a copper mine, there was a positive intervention effect measured by the JCQ, for decision latitude (0.22 points; p<0.001, η^2^=0.219), supervisor social support (0.13; p<0.004, η^2^=0.130) and coworker social support (0.1; p<0.02, η^2^=0.083).^19^ A study measuring changes in depression and suicide-related behaviours in male employees in an IT company found there was no intervention effect between baseline and follow-up in both control and intervention groups for three JCQ scales: demand (0.9, 95% CI 0.9 to 1.0; p=0.757), control (1.0, 95% CI 0.9 to 1.0; p=0.422) and support (0.9, 95% CI −0.9 to 1.0; p=0.099).^21^ However, total Hamilton depression rating scale (HAM-D) scores favourably decreased in the intervention group (1.7, 95% CI 1.3 to 1.8; p=0.001). An intervention for Dutch construction workers using the JCQ did not result in any intervention effects on social support at work (0.03, 95% CI −0.39 to 0.46), including coworker social support (0.00, 95% CI −0.21 to 0.20) or supervisor support (0.09, 95% CI −0.18 to 0.36).^22^ Furthermore, as measured by the Utrecht Work Engagement Scale, there were no intervention effects for work engagement (0.02, 95% CI −0.12 to 0.15) and the accompanying subscales (vigour 0.02, 95% CI −0.19 to 0.15; dedication 0.07, 95% CI −0.08 to 0.22) and a small negative effect for absorption (−0.09, 95% CI −1.64 to 1.46) at 3, 6 and 12 months.

### Albatross plots

**Figure 3:**
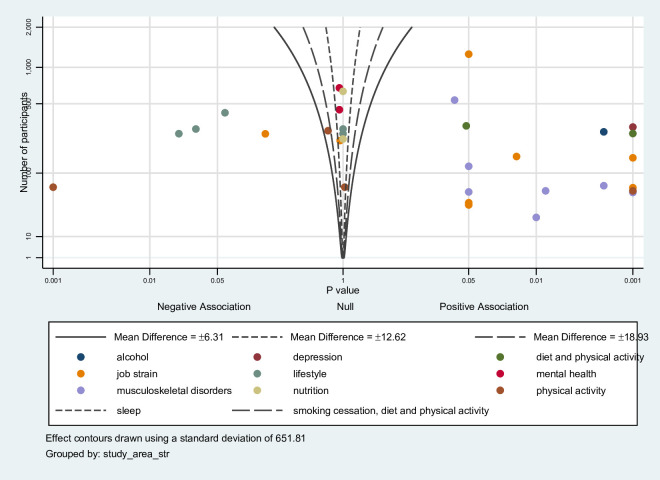
Albatross plot.

An albatross plot is presented of all studies with contours for standardised mean differences (figure 3). The studies were spread out across the plot, but there was some evidence of an improvement in employee health and well-being due to a clustering on the right-hand side. In particular, all studies with a focus on musculoskeletal disorder had a positive association, while studies focusing on lifestyle, mental health and nutrition had a negative or no association. The results for studies with low risk of bias only are provided in online supplemental figure 5. These results indicate greater clustering on the right-hand side of the plot, compared with all studies, suggesting that interventions with low risk of bias reported greater positive effect on employee health and well-being.

### Meta-analyses

Several studies evaluated interventions based on modifying blood pressure and BMI, with the outcomes being sufficiently similar to justify combined analysis (random effects).

#### Blood pressure

Ten studies reported measuring blood pressure as an outcome, but only five were sufficiently similar to combine in a meta-analysis. Based on the five studies that were included, there was little evidence of a positive effect on diastolic blood pressure (p=0.25) or on systolic blood pressure (p=0.49) (online supplemental figures 2 and 3). There was no evidence of heterogeneity in either the diastolic or systolic blood pressure findings (I^2^=1% and I^2^=0%, respectively). For studies with low risk of bias, there was also little evidence of a positive effect on diastolic blood pressure (p=0.63) or on systolic blood pressure (p=0.86), with no evidence of heterogeneity (I^2^=0%) (online supplemental figures 5 and 6).

#### Body mass index

Seven studies reported measuring BMI; however, one study was excluded as it did not report the BMI effect sizes in the paper and two studies did not include SDs. Three studies measured outcomes at 12-month follow-up^15 24 30^ and one study measured outcomes at 6 months.^39^ Based on the four included studies, there was little evidence of an effect on BMI, as shown in online supplemental figure 4, with low heterogeneity (I^2^=25%). All studies were classified as low risk of bias.

## Discussion

This systematic review was based on published literature for RCTs or non-randomised intervention group allocation reporting the effectiveness of workplace interventions aimed at improving health and well-being in male-dominated industries. A total of 35 studies met the inclusion criteria, undertaken in 14 different countries between 1998 and 2020. Given the heterogeneity across the interventions and outcome measures, meta-analysis could only be conducted for blood pressure and BMI. The sensitivity analysis indicated that studies with a low risk of bias reported larger effect sizes compared with high risk of bias studies. The main conclusion from this review is that there is some evidence that interventions specifically targeted at male-dominated industries and aimed at improving employee health and well-being can be effective. Evidence from more gender-mixed or female-dominated industries generally reports much more positive findings.^48^

It has been proposed that interventions addressing the level of work organisations or the work environment may produce more sustainable effects on the health of employees than interventions focusing mainly on individual behaviours.^49^ Five studies in this review^27 28 34 36 40^ used an organisational approach rather than addressing individual-level characteristics. In three of these studies, the intervention had a favourable effect on outcome measures, in comparison with 16 (out of 27) studies that focused on individual-level characteristics. Interventions addressing individual behaviours, that is, smoking cessation, sedentary behaviour and alcohol consumption, limited the long-term adherence to behaviour changes. This suggests that including employees alongside management in the promotion of behaviour changes provides a learning experience to understand the working environment and ultimately increase the effectiveness of the intervention.^27^

Various delivery methods were used in the interventions included in this systematic review. A systematic review which reviewed web-based interventions delivered in the workplace concluded that interventions can have positive effects post intervention on both employees’ psychological well-being and work effectiveness.^50^ A study included in this review surmised that delivering the intervention via the internet for reducing risk factors for type 2 diabetes led to a reduction in the prevalence of overweight/obesity significantly in the intervention group.^30^

In comparison with face-to-face delivery, using the internet can be more cost-effective, sustainable and potentially scalable to a wider audience. Furthermore, due to the transient nature of an employee’s working environment, particularly common within male-dominated industries, an internet intervention can be relevant to remote workers and those with non-conventional schedules.

### Limitations of the studies in the review

One of the limitations of the studies included in this review was that the majority of interventions had only relatively short follow-up up to 6–12 months. This provides little data on whether these workplace interventions have led to sustainable behaviour changes. Therefore, we were limited in assessing the long-term effects and sustainability of the interventions.

While not a selection criterion, most of the studies solely focused on quantitative analysis of the outcomes, and therefore we were unable to understand if the failure in the effectiveness was due to unsuccessful implementation or if the underlying theories used in intervention development were incorrect. The absence of information regarding intervention design, context and process in studies has previously been discussed.^51^ Only five of the studies included provided information regarding qualitative evaluation, including intervention satisfaction,^29^ intervention implementation,^41^ intervention acceptability^30^ and participant engagement.^18^ Of the 35 studies included in this review, 2 studies had a high risk of bias and 16 studies had an unclear risk of bias. Studies with an unclear risk of bias did not provide details regarding selection and performance bias, in respect to allocation concealment and blinding.

Within workplace interventions, low recruitment of participants has been a common problem.^52^ In this systematic review, the studies recorded the follow-up rate of between 50% and 94%, with six studies not providing any information regarding retention rates. For countries such as the USA, where employers directly pay an employee’s health insurance, improving health and well-being of the workforce has significant financial rewards. However, it has been suggested that long-term investment in health and job satisfaction, rather than tools for employee health and well-being, may be a more effective approach.

Workplaces provide an ideal environment to implement an intervention as employees spend more than one-third of their waking hours at work, men more than women.^53^ However, it is important to recognise that interventions do not always consider the impact of an employee’s life outside of work on their health and well-being. More employees have caring responsibilities outside of the workplace which can impact the work–life balance, as well as the wider political and economic climate, including Brexit. None of the studies included in this systematic review measured non-work-related factors as part of the intervention.

### Limitations of this review

There are several limitations to this review that should be considered. Several studies could not be included due to the definition of ‘male-dominated industry’. There also may have been missed research studies as health-related outcomes relating to employee health and well-being were often identified as secondary outcomes and therefore not always included in the abstracts. In addition, grey literature was not included in the search strategy. Searches were limited to articles that had been published in the English language, increasing the likelihood that other, non-peer reviewed studies were not included and the possibility of any language bias.

There is currently no general agreement on the definition of employee health and well-being, and as a result an absence of a shared definition. Therefore, in this systematic review the definition by Grant *et al* 54 was used, which includes three different dimensions of employee health and well-being. While this definition was selected because it explores three dimensions, some studies will be excluded from the review if they have adhered to a different definition.

### Strengths of this review

To the best of our knowledge there is no published evidence synthesis of the effectiveness of workplace interventions that aim to improve employee health and well-being specifically in male-dominated industries. This paper reviews interventions that have been conducted in workplace settings and as a result identifies real-life problems that researchers, policy makers and employers should consider prior to implementation. The studies included in the systematic review came from multiple locations across the globe, including Asia, Europe, North America and Australasia. Therefore, the results are transferable to other geographical locations.

### Further research

Future studies should consider understanding the long-term implications of adhering to workplace intervention, both for employees and employers. Most of the studies included in this review included a follow-up period of up to a year postintervention, with only three studies with a follow-up longer than 1 year. Researchers also need to consider the health economics of the intervention and the impact of changing employee health and well-being has on a business’ outcomes in both public and private sectors. Within male-dominated industries, many employees are required to work various shift patterns in transient environments. Further work therefore should explore how interventions can address these barriers in implementing a workplace intervention.

Researchers need to consider an organisational rather than individual approach. To remove additional burden, organisations need to ensure that the intervention outcomes align with their business activities and what behaviour changes they wish to prioritise. By changing the culture from the promotion of risk-taking behaviours, employees will have a greater chance of adhering to the intervention for full duration and allowing researchers to measure long-term implications.

## Conclusion

The currently available evidence indicated that interventions that aim to improve employee health and well-being in the workplace of male-dominated industries had none or only limited positive effect. Improving employee health and well-being with workplace interventions is possible, but intervention content and delivery must be considered. While the majority of interventions were based at individual level, those who engaged at multiple levels, that is, policy, environmental and individual, appeared to be more effective. This systematic review further indicated that despite the different culture within male-dominated industries compared with mixed-gender industries, workplace interventions that aim to improve health and well-being in employees can have positive outcomes.
