# Supplementary material for: Workplace interventions that aim to improve employee health and well-being in male-dominated industries: a systematic review
Source: Occup Environ Med. 2021 May 25;79(2):77–87. doi: 10.1136/oemed-2020-107314 (PMC8785069; doi:10.1136/oemed-2020-107314)
Supplement: Supplementary data [file oemed-2020-107314supp001.pdf]

Online Supplementary Figure 1: Search Strategy

Search History (26) View Saved

#	Searches	Results	Type	Actions	Annotations
1	male-dominated industr*.tw.	23	Advanced	Display Results More	
2	building industry/	3401	Advanced	Display Results More	
3	construction industr*.tw.	1758	Advanced	Display Results More	
4	manufacturing industry/	1835	Advanced	Display Results More	
5	exp coal mining/ or mining/	19674	Advanced	Display Results More	
6	agriculture/	44228	Advanced	Display Results More	
7	agriculture industr*.tw.	359	Advanced	Display Results More	
8	transport industr*.tw.	143	Advanced	Display Results More	
9	information technology/	11019	Advanced	Display Results More	
10	manufacturing industr*.tw.	1834	Advanced	Display Results More	
11	mining industr*.tw.	1120	Advanced	Display Results More	
12	information technology industr*.tw.	44	Advanced	Display Results More	
13	IT industr*.tw.	111	Advanced	Display Results More	
14	1 or 2 or 3 or 4 or 5 or 6 or 7 or 8 or 9 or 10 or 11 or 12 or 13	82215	Advanced	Display Results More	
15	workplace/	43181	Advanced	Display Results More	
16	workplace intervention*.tw.	870	Advanced	Display Results More	
17	office*.tw.	116778	Advanced	Display Results More	
18	workplace program*.tw.	206	Advanced	Display Results More	
19	15 or 16 or 17 or 18	158415	Advanced	Display Results More	
20	body mass/	453362	Advanced	Display Results More	
21	body mass index.tw.	273514	Advanced	Display Results More	
22	diet.tw.	414776	Advanced	Display Results More	
23	exercise.tw.	342601	Advanced	Display Results More	
24	fitness.tw.	83568	Advanced	Display Results More	
25	20 or 21 or 22 or 23 or 24	1229510	Advanced	Display Results More	
26	14 and 19 and 25	73	Advanced	Display Results More	

Contract

Online Supplementary Figure 2: Forest plot of the effectiveness of workplace interventions on participant's diastolic blood pressure for all studies

	Intervention			Control				Std. Mean Difference	
Study or Subgroup	Mean	SD	Total	Mean	SD	Total	Weight	IV, Random, 95% CI	Year
Kawakami 1999	76.5	11.5	81	73.8	12	77	12.4%	0.23 [-0.08, 0.54]	1999
Evans 1999	83.8	11.5	10	78.7	9.4	31	2.3%	0.50 [-0.22, 1.23]	1999
Groeneveld 2010	85.4	10.2	259	85.3	9.8	257	39.9%	0.01 [-0.16, 0.18]	2010
Limaye 2017	76.2	9.9	133	76.7	9.9	132	20.8%	-0.05 [-0.29, 0.19]	2017
Viester 2018	82.3	12.1	162	80.9	9.5	152	24.5%	0.13 [-0.09, 0.35]	2018
**Total (95% CI)**			**645**			**649**	**100.0%**	**0.07 [-0.05, 0.18]**	

Heterogeneity: Tau² = 0.00; Chi² = 4.05, df = 4 (P = 0.40); I² = 1%
Test for overall effect: Z = 1.15 (P = 0.25)

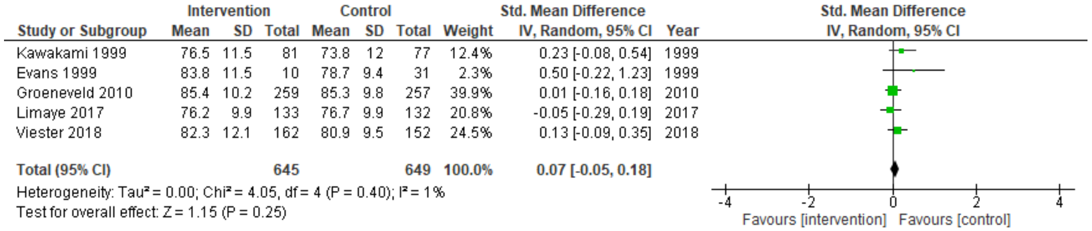

Online Supplementary Figure 3: Forest plot of the effectiveness of workplace interventions on participant's systolic blood pressure for all studies

	Intervention			Control				Std. Mean Difference	
Study or Subgroup	Mean	SD	Total	Mean	SD	Total	Weight	IV, Random, 95% CI	Year
Evans 1999	122.5	17.8	10	119.9	12.5	31	2.4%	0.18 [-0.53, 0.90]	1999
Kawakami 1999	121.4	16.4	81	118.4	13	77	12.3%	0.20 [-0.11, 0.51]	1999
Groeneveld 2010	138.1	16.6	259	137.2	16.2	257	40.2%	0.05 [-0.12, 0.23]	2010
Limaye 2017	113.1	13.4	133	114.1	12.7	132	20.7%	-0.08 [-0.32, 0.16]	2017
Viester 2018	133.9	18.4	162	133.7	13.3	152	24.5%	0.01 [-0.21, 0.23]	2018
**Total (95% CI)**			**645**			**649**	**100.0%**	**0.04 [-0.07, 0.15]**	

Heterogeneity: Tau² = 0.00; Chi² = 2.16, df = 4 (P = 0.71); I² = 0%
Test for overall effect: Z = 0.69 (P = 0.49)

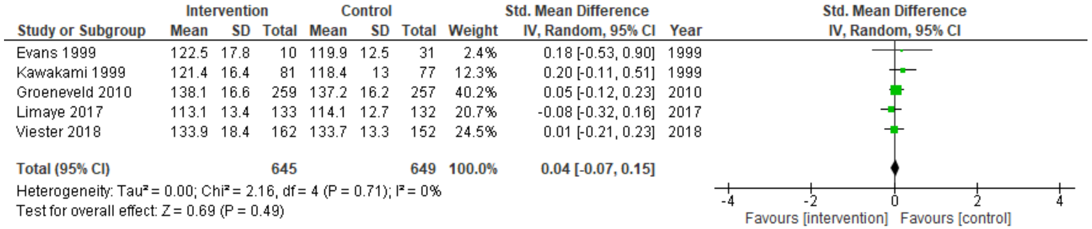

Online Supplementary Figure 4: Forest plot of the effectiveness of workplace interventions on participant's BMI for all studies

	Intervention			Control				Mean Difference	
Study or Subgroup	Mean	SD	Total	Mean	SD	Total	Weight	IV, Random, 95% CI	Year
Groeneveld 2010	28.5	3.7	261	28.5	3.9	256	39.3%	0.00 [−0.66, 0.66]	2010
Limaye 2017	26.6	3.2	133	27.6	3.7	132	28.7%	−1.00 [−1.83, −0.17]	2017
Viester 2018	27.5	3.5	127	27.9	4	129	24.8%	−0.40 [−1.32, 0.52]	2018
Matsugaki 2019	24.09	3.9	30	23.72	3.6	30	7.2%	0.37 [−1.53, 2.27]	2019
**Total (95% CI)**			**551**			**547**	**100.0%**	**−0.36 [−0.89, 0.17]**	

Heterogeneity: Tau² = 0.07; Chi² = 4.00, df = 3 (P = 0.26); I² = 25%
Test for overall effect: Z = 1.33 (P = 0.18)

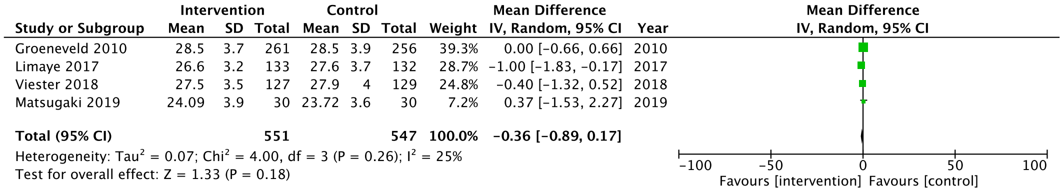

Online Supplementary Figure 5: Albatross plot for studies identified as low risk of bias

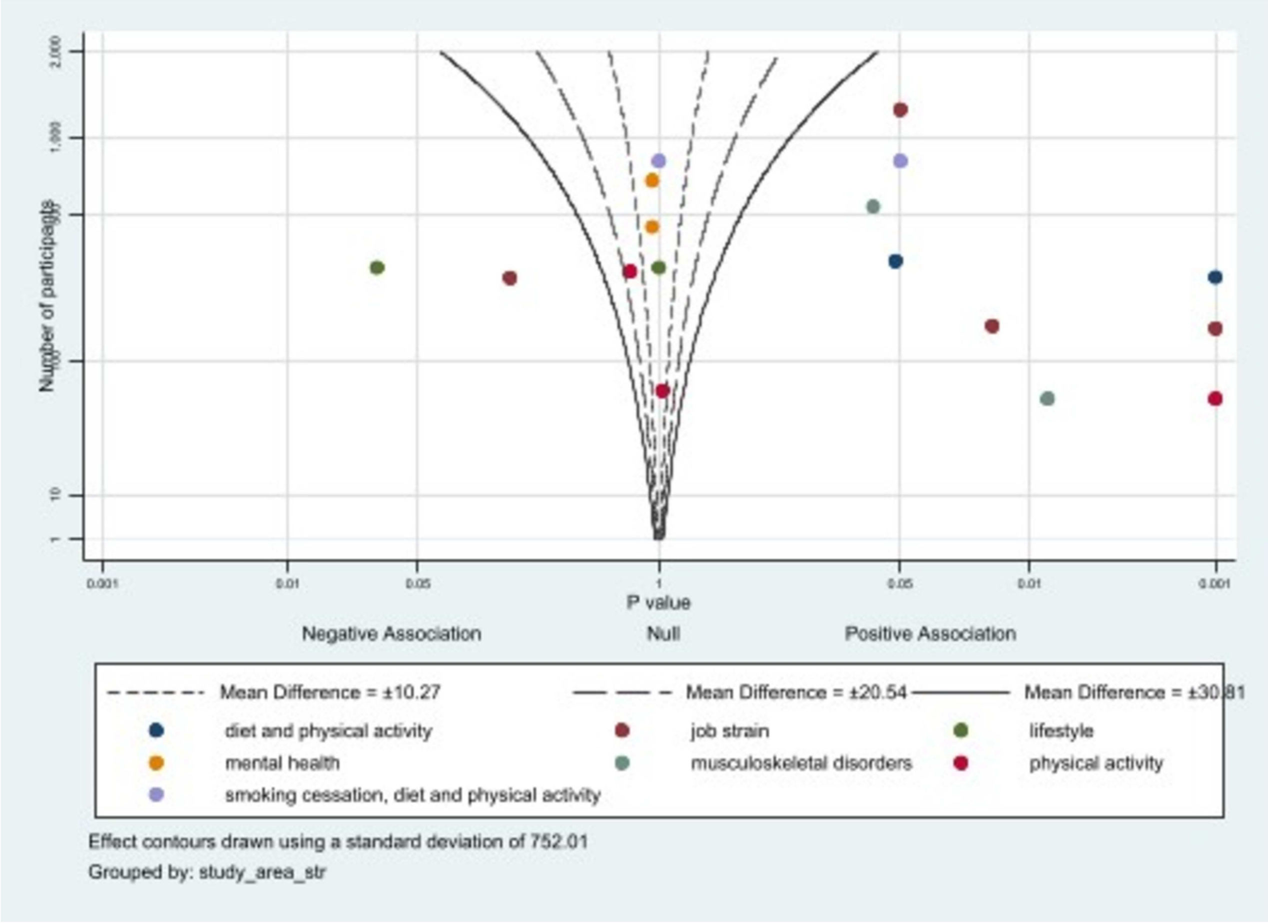

Online Supplementary Figure 6: Forest plot of the effectiveness of workplace intervention on participant's diastolic blood pressure for studies identified as low risk of bias

	Intervention			Control				Std. Mean Difference
**Study or Subgroup**	**Mean**	**SD**	**Total**	**Mean**	**SD**	**Total**	**Weight**	**IV, Random, 95% CI**
Groeneveld 2010	85.4	10.2	259	85.3	9.8	257	47.2%	0.01 [−0.16, 0.18]
Limaye 2017	76.2	9.9	133	76.7	9.9	132	24.2%	−0.05 [−0.29, 0.19]
Viester 2018	82.3	12.1	162	80.9	9.5	152	28.6%	0.13 [−0.09, 0.35]
**Total (95% CI)**			**554**			**541**	**100.0%**	**0.03 [−0.09, 0.15]**

Heterogeneity: $Tau^2 = 0.00$; $Chi^2 = 1.23$, df = 2 (P = 0.54); $I^2 = 0\%$
Test for overall effect: Z = 0.48 (P = 0.63)

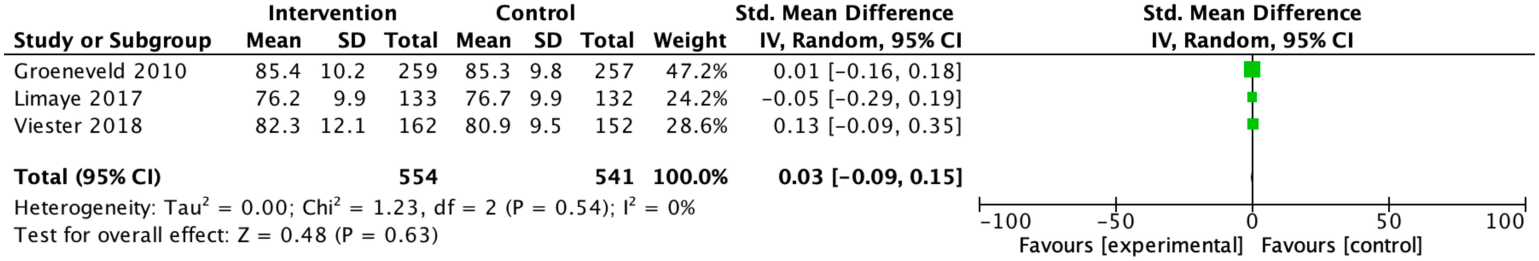

Online Supplementary Figure 7: Forest plot of the effectiveness of workplace intervention on participant's systolic blood pressure for studies identified as low risk of bias

	Intervention			Control				Std. Mean Difference
**Study or Subgroup**	**Mean**	**SD**	**Total**	**Mean**	**SD**	**Total**	**Weight**	**IV, Random, 95% CI**
Groeneveld 2010	138.1	16.6	259	137.2	16.2	257	47.1%	0.05 [−0.12, 0.23]
Limaye 2017	113.1	13.4	133	114.1	12.7	132	24.2%	−0.08 [−0.32, 0.16]
Viester 2018	133.9	18.4	162	133.7	13.3	152	28.7%	0.01 [−0.21, 0.23]
**Total (95% CI)**			**554**			**541**	**100.0%**	**0.01 [−0.11, 0.13]**

Heterogeneity: $Tau^2 = 0.00$; $Chi^2 = 0.75$, df = 2 (P = 0.69); $I^2 = 0\%$
Test for overall effect: Z = 0.18 (P = 0.86)

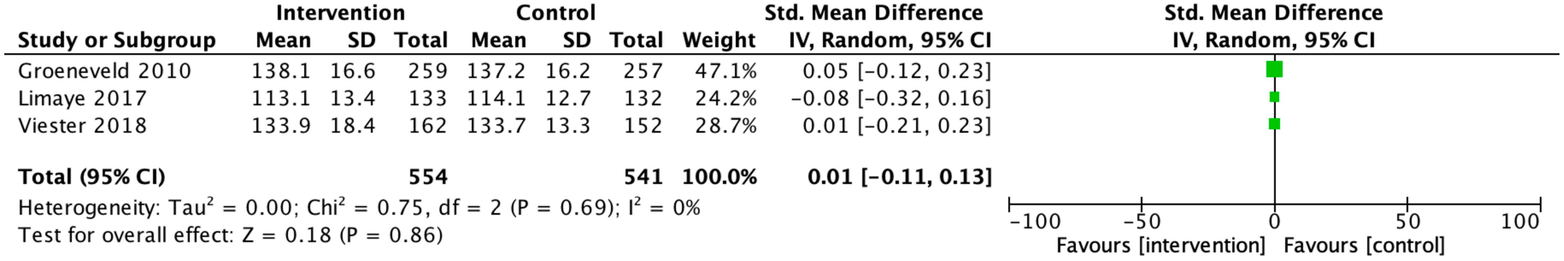

Online Supplementary Table 1: Summary of findings from included studies aiming to improve employee health and wellbeing in male-dominated industries

**Author/Year**	**Main findings**
Anderson, et al., (1999)	Differences between the three groups for fruit and vegetables (servings/day) and fat and fibre (grams/day) were not statistically significant. The control group had a lower intake of high-fat meat items versus intervention groups at 12-month follow up (p=0.03). Attitudinal changes towards less fat were important and less fat decreases cholesterol post-intervention (p<0.05).
Blake, et al., (2019)	In both intervention and control group, participant's physical activity increased significantly from baseline to post-intervention (p<0.05). However, there was a non-significant difference in the changes between the groups (p=0.70). The difference in changes in sitting hours was statistically significant between intervention and control group (p<0.01).
Braeckman, et al., (1999)	The nutrition programme significantly reduced reported total energy (p<0.05; 95% CI: -276–-8.83) and total fat intake (p<0.05; 95% CI: -2.98–-0.13) in the intervention group. Whilst there were no changes for the percentage of energy from saturated or mono-unsaturated fat, the intake of carbohydrates (p<0.05; 95% CI: 0.51–2.18) and proteins (p<0.05; 95% CI: 0.161–1.43) increased. Nutrition knowledge significantly increased in the intervention group (p<0.001; 95% CI: 1.09–1.59).
Evans, et al., (1999)	Changes in job hassles were significantly correlated to changes in systolic blood pressure (p<0.05), heart rate (p<0.05), and perceived stress (p<0.01), but not diastolic blood pressure change. Control group experienced little or no changes in job hassles or stress outcomes over the same time period, except for systolic blood pressure (p<0.025).
Faude, et al., (2015)	There was a group x time interaction for postural sway (p=0.02) with a reduction in the intervention group, but no relevant change in the control group (p<0.001). A group x time interaction (p=0.047) was found for the number of successful steps while walking backwards on the 3cm-wide beam (p<0.001) but was not significant for the 4.5cm-wide beam (p=0.44). There was no significant difference between intervention and control groups for jump height (p=0.50).

Gram, et al., (2012)	No significant changes were found in musculoskeletal pain in neck-shoulder dominant (p=0.90), low back (p=0.66) and hip to knee (p=0.77), work ability (p=0.21), productivity (p=0.28), perceived physical exertion (p=0.74) and sick leave (0.91) in the intervention group.
Gram, et al., (2012)	There was a significant difference in the estimated change in VO_{2max} of 0.4 l/min in the intervention group and 0.01 l/min for the control group (p<0.001), as well as in heart rate at a steady rate (p<0.001). There were no significant changes in BMI (p=0.55), fat percentage (p=0.37), systolic blood pressure (p=0.77), diastolic blood pressure (p=0.51), total cholesterol (p=0.56) or triglyceride levels (p=0.80).
Groeneveld, et al., (2011)	For snack and fruit intake, there was a statistically significant positive effect; β-1.9 (95% CI: -3.7–-0.02) and β1.7 (95% CI: 0.6–2.9), respectively. For snack intake, the effect was sustained at 12 months; β-1.9 (95% CI: -3.6–-0.02). For leisure time PA, the intervention was not statistically significant. At 6 months, the beneficial effect on smoking, was statistically significant; OR smoking 0.3 (95% CI: 0.1–0.7), but not at 12 months; OR 0.8 (95% CI: 0.4–1.6).
Groeneveld, et al., (2010)	In the intervention group, body weight significantly decreased at 6 and 12 months; β-1.9; 95% CI: -2.6–-1.2 and β-1.8; 95% CI: -2.8–-1.1, respectively. At 12 months, the intervention group had lost an average 1.4kg of body weight, whilst the control group had gained 0.8kg. There was also a significant change in diastolic blood pressure at 6 months; β-1.7; 95% CI: -3.3–-0.1. BMI modified the intervention effects on body weight, systolic blood pressure and HDL cholesterol, with the effects largest among the obese.
Gupta, et al., (2018)	No statistically significant overall effects on any of the outcomes were found; need for recovery (p=0.06), work ability (p=0.25), productivity (p=0.88), physical exertion at work (p=0.15), physical demands (p=0.78), physical resources (p=0.89), well-being index (p=0.60), mental health (p=0.79). There was a tendency towards an overall increased poor recovery in the intervention group, which was significant at 10- and 12-month follow-up.
Hammer, et al., (2015	For safety participation and safety compliance, mean scores were not significantly higher at 12-months in the intervention group; β=0.14 (p=0.12) and β=-0.02 (p=0.83), respectively. Mean blood pressure was significantly lower at 12-months, controlling for baseline blood pressure, age and use of medication; β=-

	2.15, p=0.038. There was no significant difference for mean SF-12 physical health scores between intervention and control group; β=-0.32, p=0.069.
Holmstrom, et al., (2005)	Thoracic and trunk flexion increased significantly in the intervention group post-intervention (p<0.001) and decreased in the control group. The stretch ability in the left and right hamstring muscles increased significantly (p<0.001) as well as in the left and right hip flexors (p<0.01). There were no significant changes in the intervention group for relative segmental flexion mobility in the cervico-thoracic spine.
Kang, et al., (2018)	In the unstable surface group, VAS, ODI and BDI scores significantly decreased (p<0.01) whilst strength and stability significantly increased (p<0.01). In the stable surface group, VAS scores significantly decreased (p<0.01) and stability, ODI and BDI scores were significantly increased (p<0.01). Between the two groups, there were significant differences in the VAS, ODI and BDI scores as well as strength and stability (p<0.01).
Kawakami, et al., (1999)	Intervention effect on GHQ score was not significant (p=0.164), although the scores significantly decreased in both the intervention and control group at follow-up versus baseline (p<0.001). Intervention effect was not significant for systolic (p=0.933) or diastolic blood pressure (p=0.314), total serum cholesterol (p=0.234) or serum triglycerides (p=0.488). There was no significant effect on sick leave, leisure-time physical activity or green vegetable intake. The intervention was significant for regular breakfast consumption (p=0.091).
Kobayashi, et al., (2008)	For men, a significant beneficial intervention effect was observed for poor physical work environment (p=0.075). For women, a beneficial intervention effect was observed for skill underutilization, supervisor, and co-worker support, and job satisfaction (p<0.05). For both genders, no intervention effect was found for sick leave (p<0.05).
Limaye, et al., (2017)	At 6 months, the intervention group had significantly greater reductions in weight (p<0.001), waist circumference (p<0.001), systolic blood pressure (p=0.012), diastolic blood pressure (p=0.033). Improvements were sustained at one year, except for systolic and diastolic blood pressure. At 12 months, the prevalence of overweight/obesity in the experimental group decreased by 6.0% and increased in the control group by 6.8% (RD 11.2%; 95% CI; 1.2–21.1; p=0.04). 98% of participants

	continued to use the virtual assistance and 96% would recommend the intervention to family and friends.
Limm, et al., (2011)	Reduction in perceived stress reactivity in the intervention group was significantly higher than in the control group; 54.5 to 50.2 and 54.5 to 52.7, respectively (f=5.932; p=0.016). No significant group x time effects were found for depression (d=0.262; 95%CI: -0.068 to 0.592 and d=0.107; 95%CI: -0.209 to 0.423) and anxiety (d=0.194; 95%CI: -0.134 to 0.522 and d=0.209; 95%CI: -0.109 to 0.527) between intervention and control groups, respectively. No intervention effect was observed in cortisol analyses.
Maes, et al., (1998)	No statistically significant effects over time were found on lifestyle variables. There were no significant differences found between the experimental and control for general stress reactions, but there was a significant difference between groups in perceived psychological demands over time (p<0.01). The intervention did not lead to significant changes over time regarding social support from supervisors and colleagues. Post intervention, absenteeism in the experimental group had decreased to 7.7% versus 9.5% in the control group.
Matsugaki, et al., (2019)	In both groups, the chair stand test improved significantly (p<0.001) following the intervention. In comparison to the control group, the intervention group had significant greater improvement in chair stand results (p<0.001). Grip strength also increased and had a significant effect observed in the intervention group (p=0.019).
McCraty, et al., (2003)	Intervention group had a means adjusted reduction of 10.6mmHg in systolic blood pressure and 6.3mmHg in diastolic blood pressure; reduction in systolic blood pressure was significant (p<0.05). Intervention group also had significant increases in positive outlook (p<0.01), peacefulness (p<0.05) and stress symptoms (p<0.05) via the POQA.
Milner, et al., (2018)	There was no significant effect on self-stigma in the intervention group. For help-seeking inhibition, shame and self-blame, there was a non-significant reduction in the intervention group at six weeks -0.03 (95% CI: -0.74–0.68), -0.19 (95% CI: -0.96–0.58) and -0.06 (95% CI: -0.50–0.39), respectively. Process evaluation suggest that participants enjoyed the program and beneficial to their mental health.

Milner, et al., (2020)	The intervention had no significant effect on suicidal thoughts (p=0.420), communication (0.056) or suicide attempts (p=0.692).
Molek-Winiarska, et al., (2018)	In the intervention group, there was a significant increase in decision latitude (f=17.36; p<0.001) and social support (supervisor f=9.00; p<0.004 and co-worker f=5.61; p<0.020) via the JCQ. For the GHQ-28, there was a significant intervention decrease effect in anxiety (f=5.28; p<0.079) and depression (f=3.95; p<0.048).
Muñoz-Poblete, et al., (2019)	The intervention had a protective effect on perceived pain intensity in upper limbs (RR: 0.62, 95% CI 0.44-0.87). There was a significant improvement in difficulty in performing work (p=0.041), difficulty in performing work as well as you would wish (p=0.021) and everyday functional difficulties in the last week (p=0.018).
Muyor, et al., (2012)	There was a significant increase in toe-touch score (p<0.01) and straight leg raise angle in both legs (p<0.01). There weren't any significant changes found in the standing postures in either experimental or control groups. There was a significant decrease in thoracic curve and significant increase in pelvic inclination were found in the toe-touch test within the experimental group (p<0.05).
Nakao, et al., (2007)	There was a significant intervention decrease effect in total HAM-D scores (p=0.0011) and individual scores of HAM-D items (suicidal thoughts, agitation, psychomotor retardation, guilt and depressed mood). There were no significant changes in JCQ scores (demand, control and support) from baseline to follow-up for both groups (all p>0.05).
Nishinoue, et al., (2012)	The average PSQI score of both groups significantly decreased and the intervention group decreased significantly more than the control group; 1.7 versus 0.3, respectively. The change in the proportion of workers with PSQI scores of poor sleep quality decreased by 23.3% in the intervention group and 11.5% in the control group; the difference between the groups was significant (p=0.08).
Oude Hengel, et al., (2012)	There were no differences found between intervention and control groups in work engagement (β0.02; 95% CI: -0.12–0.15), social support at work (β0.03; 95% CI: -0.39–0.46) and need for recovery (OR 1.17;

	95% CI: 0.66–2.07). However, at 6 months, the control group reported a small but significant reduction of physical workload (β0.18; 95% CI: 0.01–0.34).
Oude Hengel, et al., (2013)	There were no differences found between intervention and control groups in work ability (β0.02, 95% CI: -0.3–0.37), physical health status (β0.04, 95% CI: -1.43–1.35) and mental health status (β0.80, 95% CI: -0.51–2.11). There were declines in musculoskeletal symptoms (from OR 0.68, 95% CI:0.34 –1.33 to OR: 0.86, 95% CI: 0.47 – 1.57) and long-term sick leave (OR 0.44, 95% CI: 0.13–1.26), but both decreases were not statistically significant.
Pidd, et al., (2018)	No significant intervention effect was observed for risky drinking, but there was a significantly increased awareness of alcohol policy and alcohol assistance in the intervention group; p=0.001 and p=0.01, respectively. At 24 months post-intervention in comparison to the control group, for the intervention group the odds of being aware of workplace policy were 48.9% (95% CI: 29.3–88.9%) and for employee assistance were 79.7% (95% CI: 11.5–91.8%).
Rasotto, et al., (2015)	In the intervention group, there was a reduction in shoulder pain (p=0.007), as well as increases in handgrip strength (p=0.013) and back scratch (p=0.014) scores. There were also improvements in a range of movements including: shoulder flexibility (p=0.008), shoulder elevation (p=0.035), shoulder abduction (p=0.003), lateral inclination (p<0.001) and rotation of the head (p=0.002).
Umanodan, et al., (2009)	A beneficial intervention effect was found on knowledge about stress (f=32.929, p<0.001) and professional efficacy (f=3.246; p=0.074). For three items on the professional efficacy scale; ‘I can solve problems’, ‘I feel I am making an effective contribution’ and ‘I am confident at my work’, there was a favourable significant effect, however, there was no intervention effects on psychological distress, physical complaints or job performance (p>0.05).
Umanodan, et al., (2014)	There was a statistically significant group x time interaction on knowledge about stress management (f=6.028; p=0.003). Knowledge scores significant increased from baseline to 9 weeks in the intervention group (p<0.001) and significantly decrease from baseline to 19 weeks in the control group (p=0.023). There were small intervention effects on other primary or secondary outcomes at 9 and 19 weeks, but

	not statistically significant. 40% of intervention group completed the program in one or two days, instead of one per week.
Viester, et al., (2018)	At 6 months, there was a statistically significant intervention effect on body weight β-1.06 (95% CI: -1.87–-0.26; p=0.01), BMI β-0.32 (95% CI:-0.57–-0.08; p=0.01) and waist circumference β-1.38 (95% CI: -2.63–-0.12; p=0.03), but at 12 months these differences were not statistically significant. At 6 months, there was a significant increase in the percentage within the intervention group meeting public health guidelines for vigorous PA (OR 2.06, 95% CI: 1.07–3.99) and a decrease for sugar-sweetened beverages (β-2.82, 95% CI: -4.67–-0.97).
Zebis, et al., (2011)	There was a significant decrease in neck pain intensity in the intervention group versus control group (-0.6; 95% CI: -1.0–-0.1) as well as decreases in shoulder pain intensity (-0.2; 95% CI: -0.5–0.1).

Beck Depression Inventory **(BDI)**; Body Mass Index **(BMI)**; General Health Questionnaire **(GHQ)**; General Health Questionnaire – 28 version **(GHQ-28)**; Hamilton Depression Rating Scale **(HAM-D)**; High Density Lipoprotein **(HDL)**; Job Content Questionnaire **(JCQ)**; Oswestry Disability Index **(ODI)**; Personal and Organisational Quality Assessment **(POQA)**; Physical Activity **(PA)**; Pittsburgh Sleep Quality Index **(PSQI)**; Risk Difference **(RD)**; Short Form Questionnaire – 12 version **(SF-12)**; Visual Analog Scale **(VAS)**
